# Supplementary material for: Short-term hormonal modulation with mifepristone does not induce oncogenic changes in the endometrium of BRCA1/2 pathogenic variant carriers
Source: Commun Med (Lond). 2026 Feb 11;6:150. doi: 10.1038/s43856-026-01412-0 (PMC12996395; doi:10.1038/s43856-026-01412-0)
Supplement: Supplementary file 2 — Supplementary Information [file 43856_2026_1412_MOESM2_ESM.pdf]

Supplementary Information

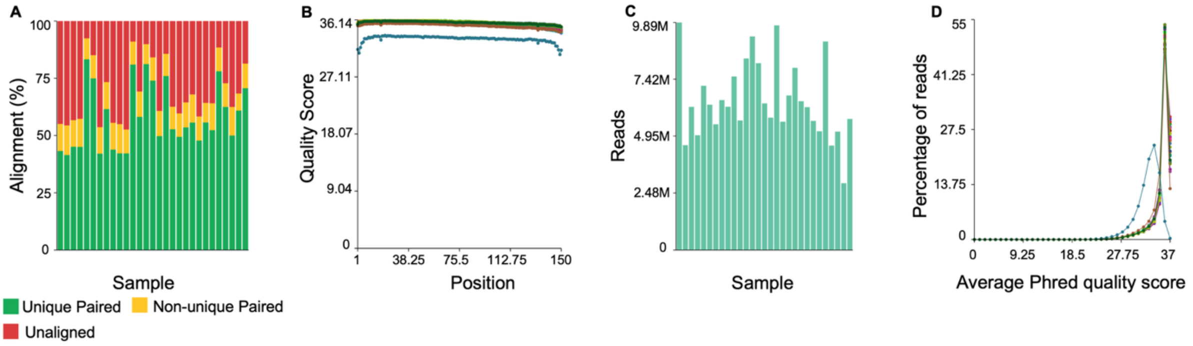

Supplementary Figure 1. RNA seq data quality control.
A. Percentage of alignment to the human genome. B. Average base quality score. C. Total reads for each sample. D. Average Phred quality score.

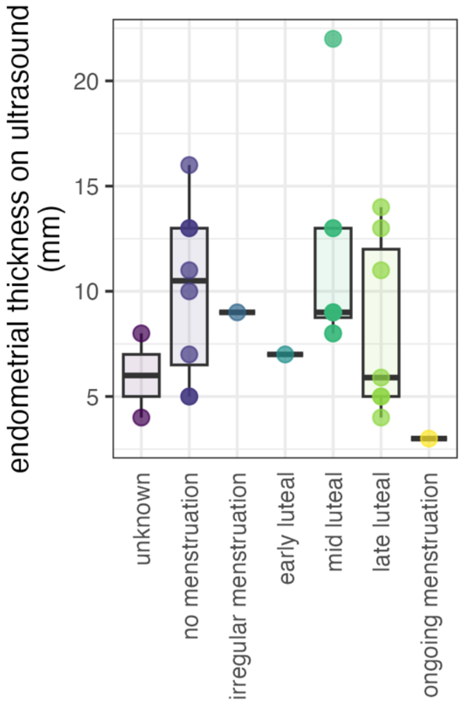

Supplementary Figure 2. Endometrial ultrasound thickness by luteal phase in the current study.

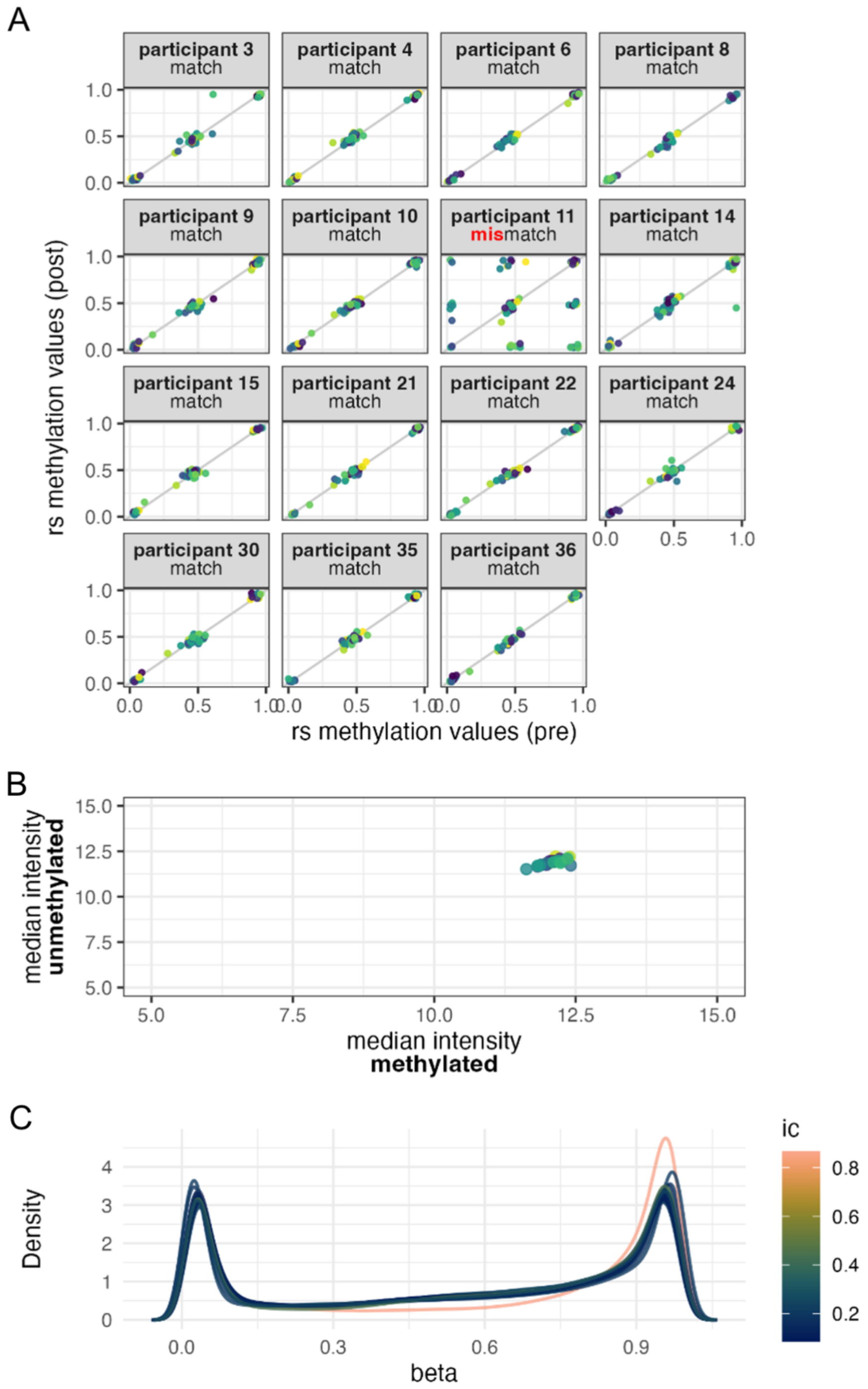

Supplementary Figure 3. In-depth overview of quality control of DNA methylation data generated using the Illumina Human Methylation EPIC array. A. Analysis of rs (single nucleotide polymorphism) probe analysis reveals mismatches in SNPs of one participant that was excluded for downstream analysis. B. Median unmethylated and methylated array intensities. C. Beta distribution densities reveal one outlier, driven by a different cell type composition.

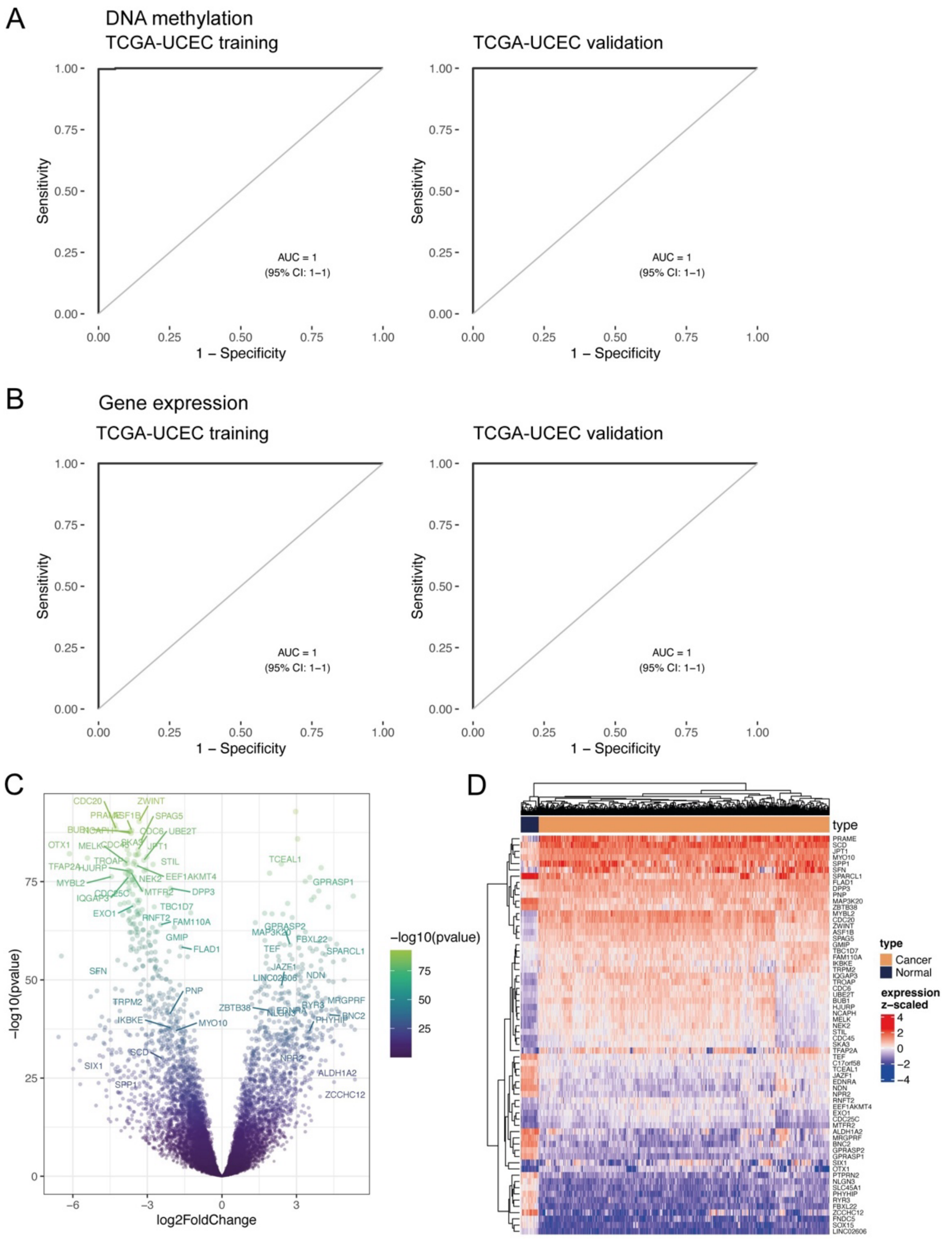

Supplementary Figure 4. DNA and RNA-based endometrial cancer classifiers. A. AUC of the DNA methylation-based Endometrial Cancer tissue index (TCGA-EC methylation index) in training and testing sets. B. AUC of the gene expression-based Endometrial Cancer tissue index (TCGA-EC expression index) in training and testing sets. C. Volcano plot of differentially expressed genes between normal endometrial and endometrial cancer tissue in the TCGA training set. Labels indicate genes included in the final classifier (5 genes are missing due to overlaps). D. Heatmap plot of row-scaled gene expression values of genes included in the expression-based TCGA-EC index in the training set shows strong differences in expression.

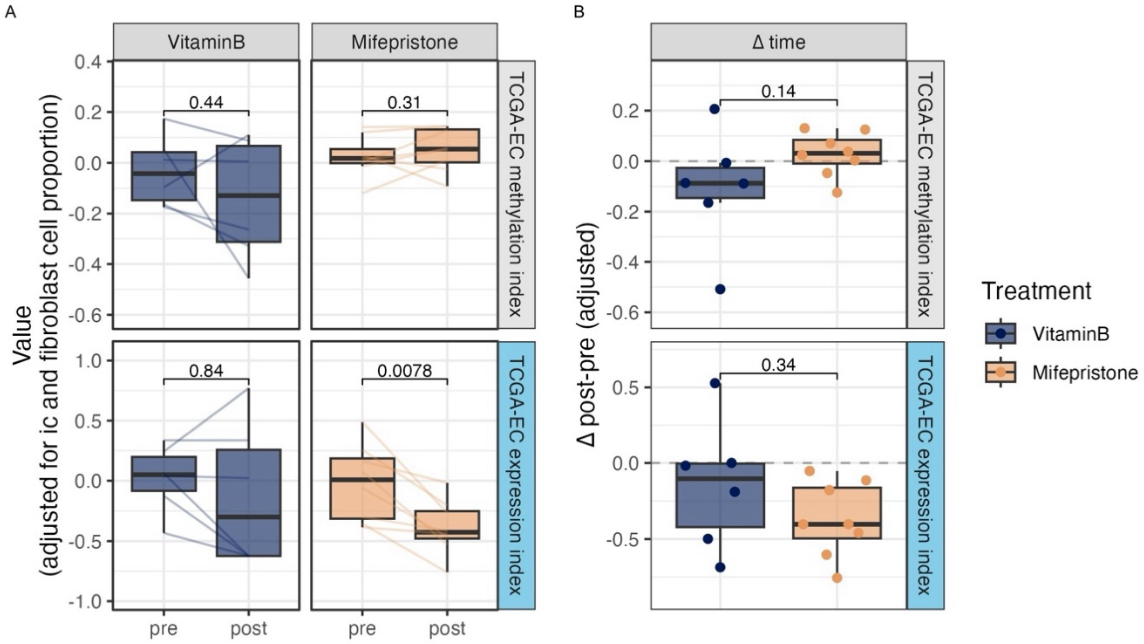

Supplementary Figure 5. Changes in cancer-indicative DNA methylation and gene expression biomarkers upon mifepristone treatment after adjustment for age and fibroblast proportion. A. Paired pre-and post-values of cancer-indicative biomarkers associated with biomarkers of endometrial cancer based on DNA methylation or gene expression, trained using TCGA data (TCGA-EC scores). Values were adjusted for age and fibroblast proportion. p values are derived from paired Wilcoxon tests. B. Change in biomarker values from baseline (Δ time) in Vitamin B and Mifepristone-treated individuals. Values were adjusted for age and fibroblast proportion. p values derived from unpaired Wilcoxon tests comparing Mifepristone to Vitamin B.

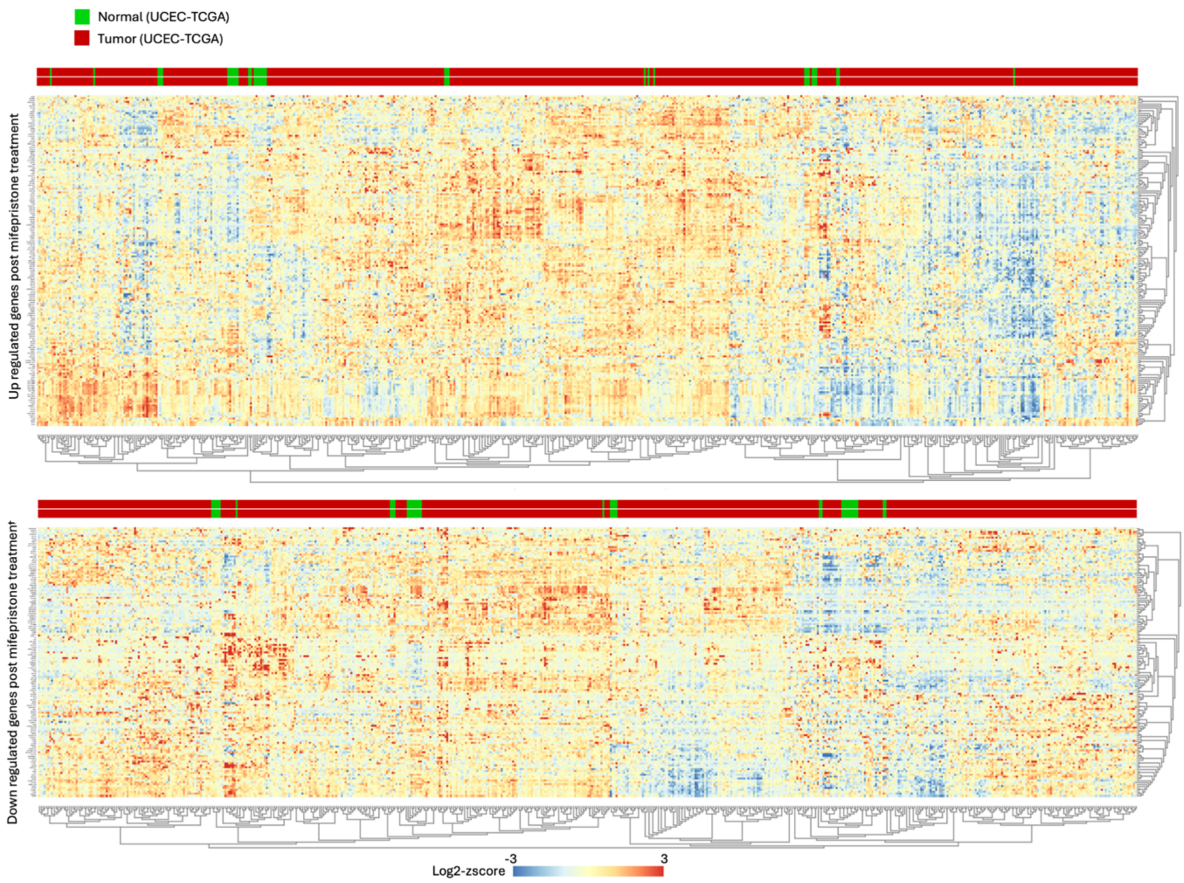

Supplementary Figure 6. Heatmap of gene expression in UCEC-TCGA data comparing normal and cancerous tissues. The genes examined are the differentially expressed genes (DEGs) between post- and pre-Mifepristone treated groups. a Upregulated DEGs. b Downregulated DEGs. DEGs in the TCGA-UCEC project were analyzed and visualized using the R2 genomic analysis and visualization platform.

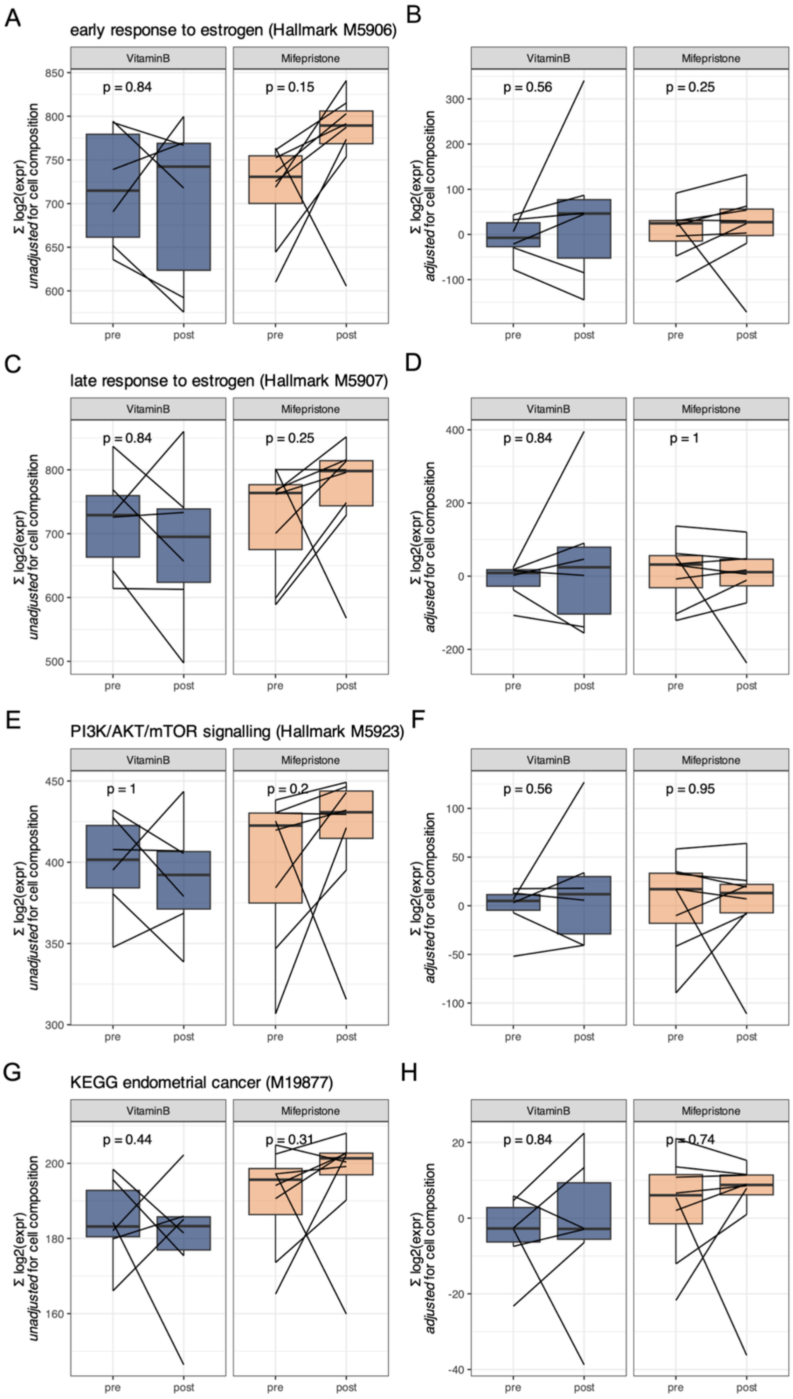

Supplementary Figure 7. Changes in endometrial cancer hallmark gene expression patterns upon mifepristone or control treatment. A & B, Sum of expression levels in genes related to early response to oestrogen, where A. represents unadjusted values, and B. shows values adjusted for cell composition (using methylation data). C & D, Sum of expression levels in genes related to late response to oestrogen, with C. unadjusted and D. adjusted for cell composition. E & F, Sum of expression levels in genes related to the PI3K/AKT/mTOR signalling pathway, with E. unadjusted and F. adjusted for cell composition. G & H, Sum of expression levels in genes involved in KEGG-endometrial cancer signalling, where G. is unadjusted and H. is adjusted for cell composition.

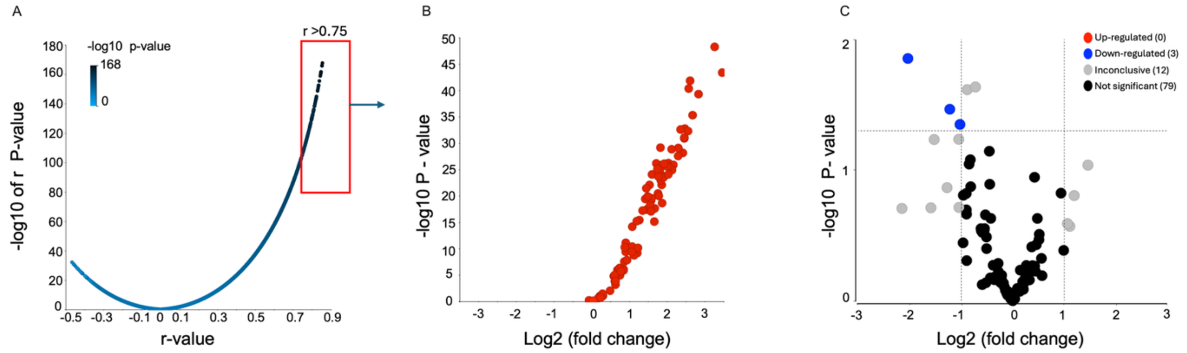

Supplementary Figure 8. BRCA related genes in endometrial carcinoma.

A. Analysis of BRCA-related genes in UCEC using the R2 Genomics analysis and visualization platform. Genes (89 genes) with positive correlation (within the red square) were selected for further analysis. All positively correlated genes had an r-value greater than 0.75. No significant negative correlations were detected. B. Comparative analysis of the selected genes from (a) showing upregulated genes in cancer tissue compared to normal tissue. Out of 89 genes, 79 showed significant upregulation (p-value $\leq$ 0.05) in endometrial carcinoma (EC) tissue compared to normal endometrium. C. Expression patterns of the 89 selected genes in endometrial tissue following mifepristone treatment. None of the tested genes were significantly upregulated (indicated by red dots if present). Three genes were significantly downregulated (blue dots), and the remaining genes were not significantly affected by mifepristone treatment.
