# Supplementary material for: Short-term hormonal modulation with mifepristone does not induce oncogenic changes in the endometrium of BRCA1/2 pathogenic variant carriers
Source: Commun Med (Lond). 2026 Feb 11;6:150. doi: 10.1038/s43856-026-01412-0 (PMC12996395; doi:10.1038/s43856-026-01412-0)
Supplement: Supplementary file 3 — Description of Additional Supplementary files [file 43856_2026_1412_MOESM3_ESM.pdf]

Description of Additional Supplementary Files

Supplementary Data 1: Patient information and BRCA status

Supplementary Data 2: CpG Coefficients for the ridge-penalized methylation index using the top 8,000 features

Supplementary Data 3. Coefficients of the 64-Gene TCGA-EC expression index distinguishing normal and cancerous endometrial tissue

Supplementary Data 4. Expression patterns of BRCA-associated genes in EC and response to mifepristone treatment

Supplementary Data 5. Numerical data underlying figures
